# Comparison of fracture resistance of implant-supported fixed prothesis substructure materials with different cross-sectional geometry

**DOI:** 10.1186/s12903-025-06033-y

**Published:** 2025-04-26

**Authors:** Oğuzhan Yılmaz, Ayşegül Göze Saygın, Giray Bolayır

**Affiliations:** Faculty of Dentistry - Department of Prosthodontics, Sivas Cumhuriyet University, Sivas, Turkey

**Keywords:** CAD/CAM, Fiber reinforced composite, PEEK, Thermomechanical ageging, Zirconia

## Abstract

**Background:**

The aim of this study was to compare fracture resistance after thermomechanical ageing of prosthetic substructure materials with different connector designs.

**Methods:**

Three different prosthetic substructure materials were used in this study: (1) GroupZir: **(**Zirconia, Fusion ceram, Turkey), (2)GroupPEEK (PEEK, Whitepeaks, CopraPeek, Essen, Germany), (3)GroupFRC (Fibre-reinforced composite, Trinia, Bicon Implant, Rep. of Ireland). A total of 72 implant-supported prosthesis triangular, square, and oval connector designs were created between 2nd premolar and mandibular 2nd molar teeth. After adhesion to the implant abutments with resin cement (Pentron breeze, Kerr), the samples were applied with dynamic loading and thermomechanical ageing (120,000 cycles,120 N,5–55 °C). Fracture resistance values were obtained with a universal test device and SEM images were analysed. The analyses were performed with Two-Way ANOVA and the Tukey test (SPSS 23.00).

**Results:**

Both the material and the connector type were found to affect the fracture resistance (F = 8.354, *p* < 0.05). The highest fracture resistance value was obtained from the triangular shape in GroupZir(3200 ± 91.05) and the lowest from the oval connector design of GroupPEEK material (2410 ± 157.23). Statistically significant differences were determined in the different connector designs of GroupZir(*p* < 0.05). In the comparisons made according to connector design, a significant difference was obtained between GroupZir and GroupPEEK and between GroupZir and GroupFRC. Deformations were observed in the fracture pattern of the Group Zir samples and deformations in the form of rupture were seen in the GroupPEEK and GroupFRC material samples.

**Conclusions:**

The study results demonstrated that the fracture resistance of zirconia, PEEK, and FRC restorations over 3-unit implants with different connectors is affected by connector design. All the materials were seen to be comparable in respect of the forces formed in chewing dynamics.

## Background

There are currently various treatment options for the replacement of missing teeth. Generally, good mid and long-term results have been proven for fixed dental prostheses (FDPs), similar to prostheses supported by teeth [1, 2]. It has also been seen that dental implants used in the treatment of missing teeth provide reliable support for FDP restorations. Moreover, for economic reasons in treatment planning, and because of poor existing bone quality and the size of the gap without teeth, the area without teeth can be rehabilitated with a 3-unit FDP supported by two implants [3].

The increased aesthetic requirement in dentistry has led to the development of metal-free FDPs. Restorations not containing metal are produced using computer-assisted design (CAD)/computer-assisted manufacturing (CAM) methods, which provide technical sensitivity, ease-of-design and manufacture, and digital workflow [4]. For the production of restorations matching the dental colour, there is a wide range of materials of which blocks or discs can be processed with CAM, primarily resins, zirconia, reinforced ceramics, or resin-infiltrated ceramics [5]. In addition, high-performance polymers such as polyetheretherketone (PEEK), polyetherketoneketone (PEKK), and fibre-reinforced composite resin (FRC), are now marketed for the production of FDPs [4].

The selection of the substructure is an important criterion in respect of the stresses transferred to the implant-abutment and peri-implant regions in implant-supported prosthetic restorations [6]. Zirconia is a dental ceramic of polycrystalline structure with the highest fracture resistance in current use. However, because of the limited glass content, the low translucency can lead to aesthetic problems, and because of the high elastic module there may be biomechanical problems in the supporting and antagonist teeth [5]. Zirconia, which is used as an alternative to Cr-Co substructures, is resistant to deformation because of the high Young modulus (200 GPa). However, there is concern about its use for restorations in the posterior region because of the high elasticity modulus [7]. In addition, as the chemical stability has not yet been fully proven, the clinical behaviour of monolithic crowns has not been sufficiently documented [8].

PEEK and PEKK are two members of the high-performance, semi-crystalline material polyaryletherketone (PAEK) family, which are often used in dentistry. PEEK can be produced with CAD/CAM technology and is used in dentistry in fixed-unit prostheses [9], implant-supported fixed prostheses, and mobile prostheses [10, 11]. PEEK is used in medical treatments as a bioinert and biocompatible material because of its chemical structure [12] PEEK has been used as a substructure material in fixed prosthetic restorations because of its shock-absorbing property and is recommended as substructure material because its brown colour is not sufficiently aesthetic [13].

The FRC, Trinia™ (BICON, Boston, MA, USA), which has been presented on the market in disc or block form, can be used as a substructure material in prosthetic rehabilitations with CAD/CAM technology. Used as an alternative to traditional methods, Trinia has high flexural strength and a flexural elastic modulus similar to that of dentin, and can be used in metal-free restorations [14, 15]. Despite a minimum CAD/CAM processing period, the bending and compressive resistance of Trinia is high. Trinia can be produced extra-orally or intra-orally, and it offers a high level of comfort during use because of its lightness. Moreover, new-generation FRC materials have been proven to have mechanical properties equivalent to those of PEEK, PEKK, and zirconia [16, 17].

There is no single form of fixed prosthetic restorations. The contours have a complex combination of several convexities and concavities depending on the alignment and geometry of the teeth. The connecting area is narrow because of biological and aesthetic reasons, and these areas in 3-unit FDPs have stress concentrations according to the mean stress levels in the other areas of the prosthesis [18]. FDPs which have a small gingival embrasure are exposed to high stress concentrations during loading in the connector area compared to FDPs with a large embrasure diameter. Consequently, the dimensions and especially the connectors of the FDP must be sufficient in general to provide optimal clinical performance. However, there is no clear evidence in the literature of what are sufficient connector dimensions and there are different variations related to this [19, 20, 21]. In stressful areas such as posterior FDPs where occlusal height is reduced, changing the connector design may play an important role in increasing the fracture burden [14].

There is a scarcity of evidence related to how connector design affects current substructure materials. The aim of this study was to compare the fracture resistance of connector designs in different prosthetic substructure materials used in fixed prosthetic rehabilitation. The null hypothesis of the study was that different connector designs would not change the fracture resistance of the substructure materials.

## Methods

Using a specifically prepared device, two implants of Grade IV titanium, 4.1 mm x 10 mm in size (Bilim Implant, Turkey), were placed parallel within autopolymerised acrylic resin (Blau acryl, Republic of Ireland). The distance between the centres of the two implants was 15 mm, as the average value between the lower 2nd premolar and 2nd molar. Flat titanium abutments of 5 mm diameter and 1 mm gingival height, as abutment to a 3-unit cemented restoration, were cleaned with an ultrasonic cleaner (Euronda Ultrasonic Energy, Vicenza, Italy) [22], then torque was applied with 25 N(N) torque in accordance with the manufacturer’s instructions. To prevent anterior loading loss, the torque procedure was repeated after 10 min. All the implants were embedded in this model.

A total of 72 3-unit FDPs were formed, based on the lower 2nd premolar and lower 2nd molar teeth. The samples were separated into 3 main groups of 24 in each group according to the material used; (1) GroupZir: (Zirconia block, Fusion ceram, Turkey), (2) GroupPEEK (Whitepeaks, CopraPeek, Essen, Germany), (3) GroupFRC (Trinia, Bicon Implant, Rep. of Ireland).

After the application of scanning spray on the model formed (Whitepeaks CALIDA, Germany), data were recorded by scanning with the laboratory CAD system (dwos 7 series Dental Wings, Straumann Group Band, Basel, Switzerland), which can perform three axis scanning [23].

The substructures were designed in anatomic form supported by a shaft 3 mm in height on the lingual and proximal surfaces. Crown height was defined as 15 mm, and the substructure material was designed in standard tesselation language (STL) format to be thickness of at least 1 mm and the cement cavity of 50 μm [24]. The data were then transferred to the CAM unit.

**Fig. 1:**
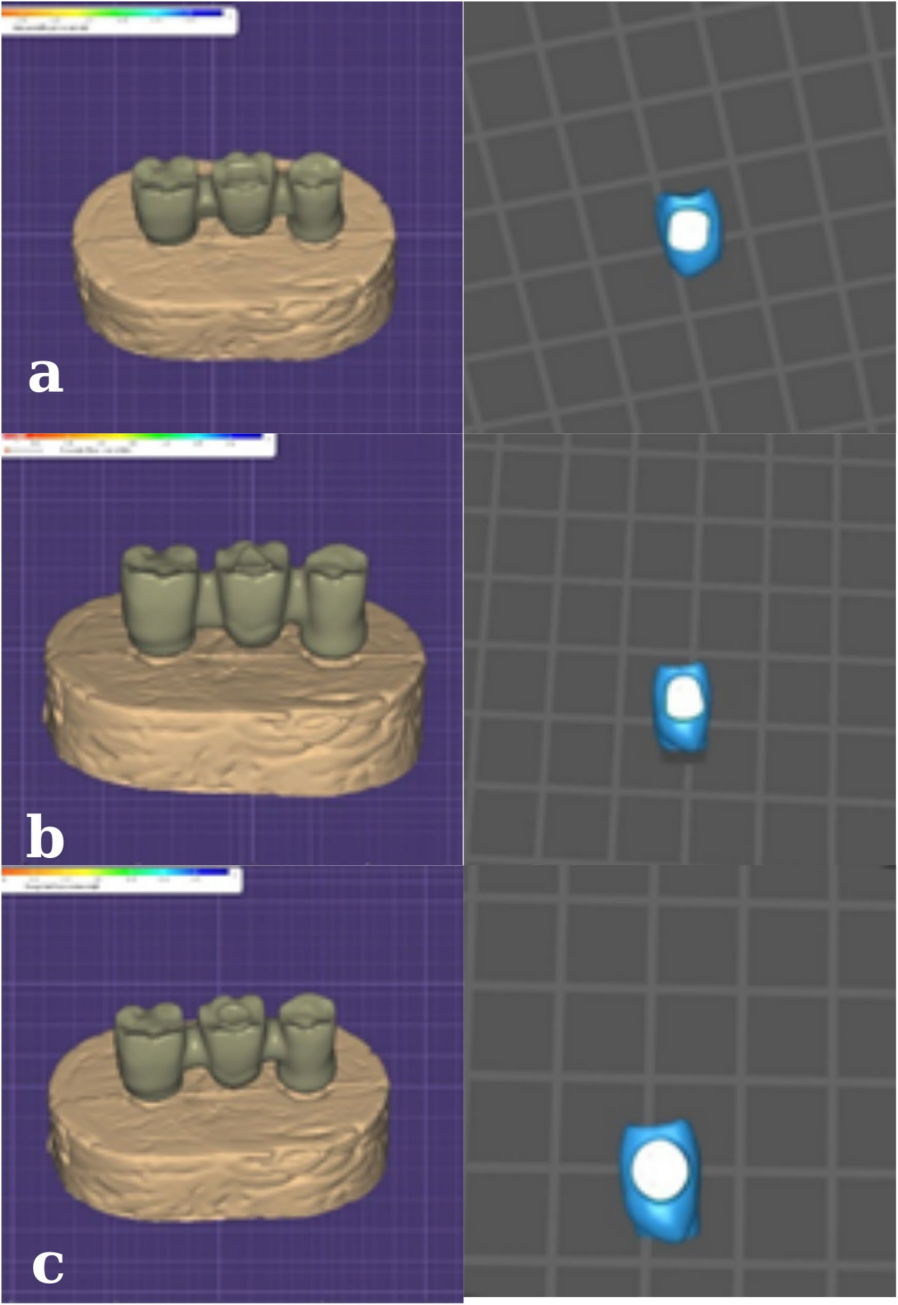
Digital images in the buccolingual plane of cross-sectional connector designs of the different substructure materials. **a**. GroupZir **b**. GroupPEEK **c**. GroupFRC **d**. square **e**. triangular **f**. Ovoid

The connector designs of the substructure materials used were designed in 3 different forms in the digital environment (Figs. 1a, b,c). With the connector area of at least 12 mm^2^, triangular, square, and ellipsoid connector designs were made.

After covering the abutments with teflon bands, the prefabricated titanium abutments were abraded with 110 μm aluminium oxide particles under 2.5 bar pressure. The inner surfaces of the crowns were washed and dried, then applied with silane (UltraDent Products GmbH, Cologne, Germany) for 30 s. The self-adhesive resin cement, Breeze (Pentron Clinical, USA), was mixed according to the manufacturer’s instructions and applied under 10 N loading.

Thermomechanical ageing was performed in an 8-chamber, double-axis chewing simulator (SD Mechatronik Chewing Simulator CS-4 Willytech, Munich, Germany). The samples were left for 48 h at 37 °C then the ageing procedure was performed under 120 N force at the antagonist end with 6 mm diameter steel ball bearings (1.2 Hz, 1.2 × 10^6^ cycles). Mechanical loading on the vertical axis was applied at movement distance of 6 mm with vertical axis speed of 55 mm/sec, and on the horizontal axis at 0.3 mm movement distance at a speed of 30 mm/sec.

After the dynamic loading, the samples were examined under a stereomicroscope (Zeiss, Germany) and no failures were observed. For the fracture resistance test, the samples were placed in a Universal Test Device (Lloyd LRX, Lloyd Instruments ltd., Hampshire, UK) under a static load and the test was performed with a stainless steel tip 5 mm in diameter so the pontic was in contact with the central fossa. Compressive tip force was applied to the samples in the test device with 0.5 mm/min head speed until the first fracture or failure. The fracture resistance value of each sample was recorded on the computer software program and graphic data were obtained.

### Statistical analysis

Effect size was calculated using G Power 3.0.10 program (Kiel University, Germany) based on the mean fracture resistance findings of Nazari et al., and Cohen’s d of 3.154 was accepted as sufficient for statistical significance [23]. For the statistical analyses, variance analysis was performed using the t-test and Two-Way ANOVA. A value of *p* < 0.05 was accepted as the level of statistical significance.

## Results

No complications such as screw loosening or fracture of the screw or crown were seen in any of the samples. After the ageing procedures applied to the samples, the survival rate was 100%. The mean and standard deviation (sd) values are shown in Table 1. At least 24 samples were needed as 3 for each of the main groups, and 0.05 type 1 error and 99% power were calculated.

**Table 1:** The fracture resistance values of the substructure materials with different connector designs

	Triangular (Mean ± sd)	Square (Mean ± sd)	Ovoid (Mean ± sd)	*p*		
GroupZir	3200 ± 91.05^a, b,A, B^	2920 ± 428.69^A^	2700 ± 267.29^B^	0.001*	F = 3.763 *p* < 0.02*	
GroupPEEK	2640 ± 305.47^a^	2650 ± 301.09	2410 ± 157.23	0.6		
GroupFRC	2680± 359.51^b^	2670± 291.41	2630 ±160.80	0.3		
p	0.001*					
Interaction Materials*Connector	F = 8.354 *p* = 0.004*					

*Statistical significance

**Capital letters indicate horizontal differences, and lower case letters indicate vertical differences

**Fig. 2:**
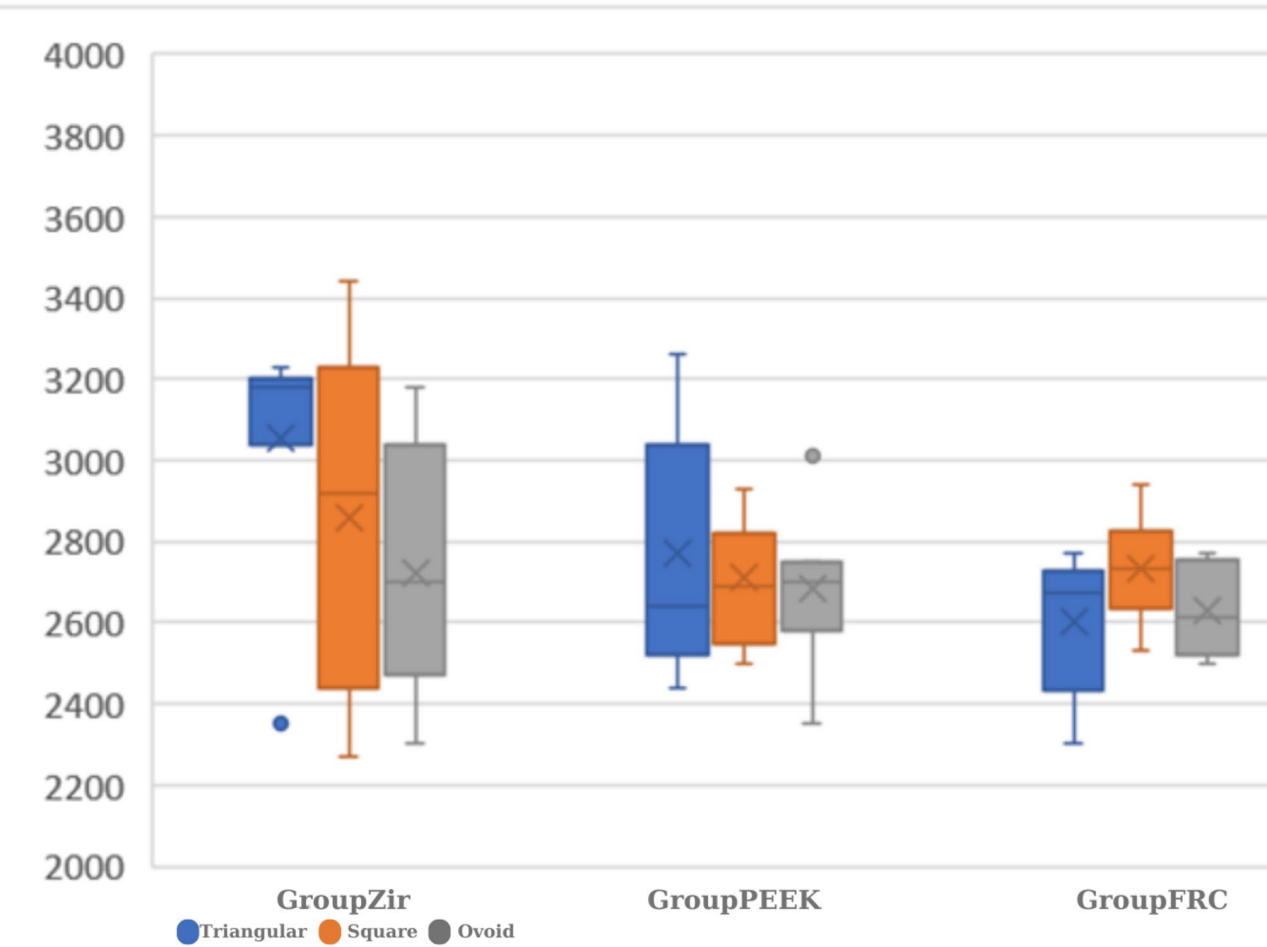
Comparisons of the fracture resistance values of the substructure materials

The highest fracture resistance values were obtained for the samples in Group Zir; triangular: 3200 ± 91.05 N > square:2920 ± 428.69 N > ovoid:2700 ± 267.29 N (Fig. 2). The differences between triangular and square and between triangular and ovoid were determined to be statistically significant (*p* < 0.05). No significant differences were seen in the GroupPEEK and GroupFRC samples, with data in the order of triangular > square > ovoid (*p* > 0.05).

In the analysis of the different connector designs according to material, the triangular shaped connectors showed statistical significance (*p* < 0.05). No significant difference was found between GroupZir and GroupPEEK or between GroupZir and GroupFRC.

**Fig. 3:**
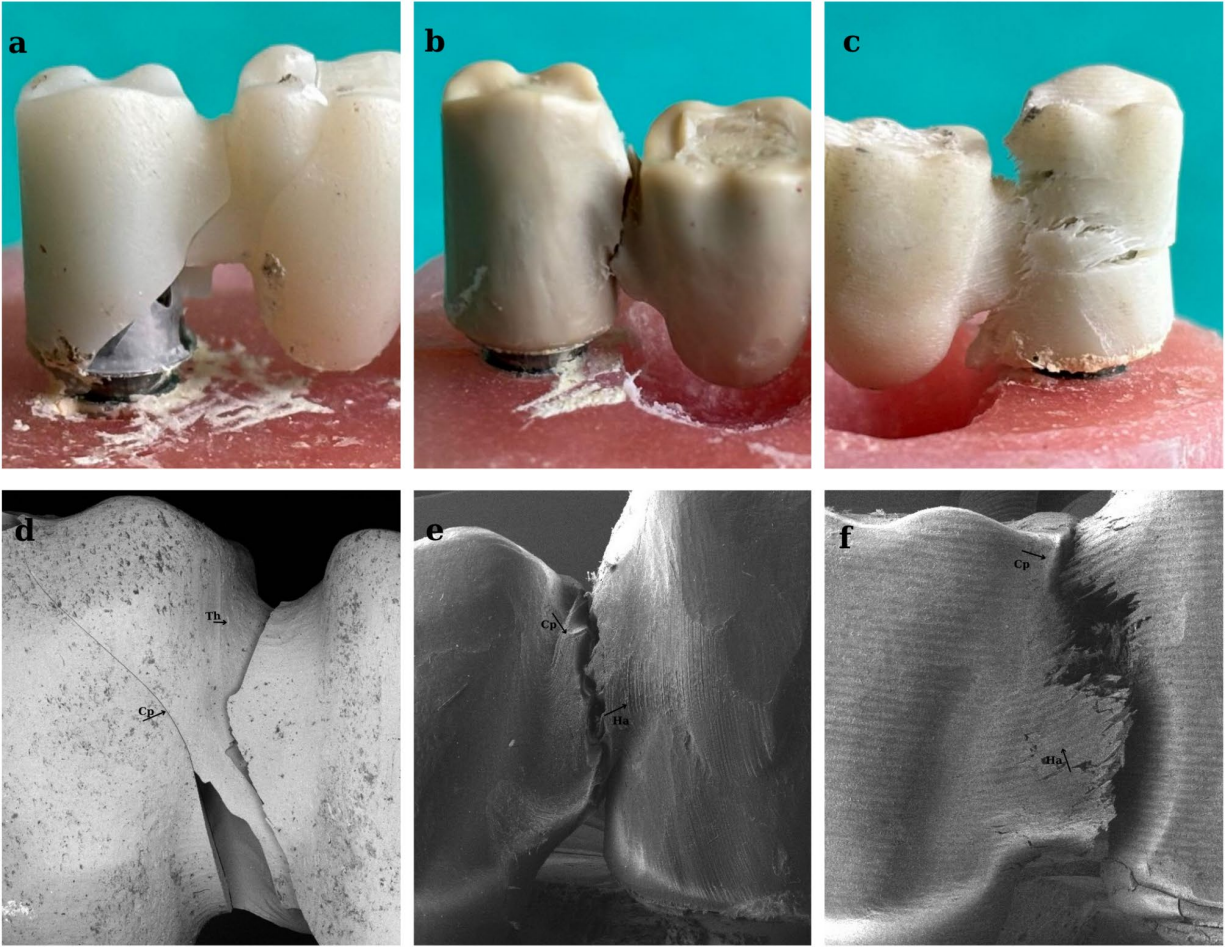
Images of the fractures in the different substructure materials Fracture line of GroupZir(**a**), GroupPEEK(**b**), GroupFRC(**c**); SEM images of GroupZir(**d**), GroupPEEK(**e**), GroupFRC(**f**) FDP specimens after compression tests. Cp: crack propagation, Ha: hackles, Th: twist

As can be seen in Fig. 3, the fracture line was clearly observed in the zirconia material (Fig. 3a), As the fragility of the material increased, so it tended to break in the weakest zone. In ductile materials such as PEEK and FRC, plateau area is formed before permanent deformations of the material occur under compressive stress. Plateau areas are the second stage and vary depending on the ductility of the material. Deformation hardening occurs in the third stage due to increased stress. This results in increased resistance of the prosthetic material known as the energy absorption limit (toughness) [19]. In GroupPEEK and GroupFRC, ruptures were seen after the occurrence of mass bending due to elastic deformation (Figs. 3b, c).

## Discussion

The fracture resistance of prosthetic substructure materials is important for the success of the treatment. The results of this study showed significant differences in the resistance strength of different connector designs (triangular, square, ovoid) of three different prosthetic substructure materials (zirconia, PEEK, FRC). Therefore, the null hypothesis of the study was rejected.

The highest stress concentrations in FDPs occur in the connector area during loading. As ceramics are extremely sensitive to tensile forces, it is important to reduce these types of stress concentrations in the gingival section of the connector where tensile forces often occur.

Although higher fracture loading values were seen for Zirconia, used as one of the substructure materials in this study, more catastrophic fractures were seen. Most fractures in zirconia FDPs are brittle fractures at the connector level [25]. This is because zirconia has a high elastic modulus (200–220 GPa), and an extremely low fullness modulus, and is stiffer and more fragile. Therefore, the relatively more fragile structure of this restorative material exhibits poorer flexibility and resistance balance to resist destructive fracture energy [8]. In most cases, fracture starts in the gingival region of the connector and extends obliquely to the pontic occlusal region. Demirci et al. reported that stress concentration in the connector surface of zirconia increased the probability of fracture during function [25].

Ceramic FDPs are extremely sensitive to tensile stresses and connector areas are accepted as the region most predisposed to fracture because of the high stress concentration. Moreover, clinical applications require that in addition to resistance to chewing forces, an ideal connector should provide acceptable aesthetics and should allow sufficient hygiene. Therefore, the geometry and dimensions of the connector cross-section may require personalisation according to the characteristics of the patient [26, 27].

The fracture resistance of FDPs can depend on several variables. Therefore, in the selection of tooth or implant upper prosthetic material, especially in the posterior region, the biomechanical properties of the material come to the fore. Although chewing forces vary together with patient-related factors, they have been determined to be mean 597 N in females and 847 N in males in the posterior region [27]. The threshold level for posterior FDPs shoud be at least 500 N [25]. Fracture resistance > 1000 N is recommended for ceramics as posterior FPD to be abe to obtain a better clinical performance [15, 25].

It has been reported that 3-unit PEEK FDPs show plastic deformation at 1200 N and fracture loading of 1383 N, and can undergo plastic deformation without complete fracture of the PEEK restorations [17, 28]. Yilmaz et al. also showed that high-performance polymers such as PEEK, PEKK, and FRC had mechanically more ductile behaviour than zirconia under loading forces [17]. According to the current study findings, rupture type failure was observed in the PEEK and FRC materials.

Mahmood et al. examined the fracture resistance of connector designs of CAD/CAM ceramics with different pontic numbers, and concluded that more pontics further reduced fracture resistance by 45% [29]. In 3-unit restorations, it has been stated that FRCs can be used as an alternative to zirconia and metal-supported restorations because of their mechanical properties and lightness [24]. When compared with conventional materials, it has been shown that FRC substructure restorations are a promising material as they act like a stress shield to minimise peri-implant bone loss [30].

Trinia, which is an FRC, is a CAD/CAM material that is light, can be processed and has high bending and pressure resistance. Ewers et al. showed that this polymer had clinical success comparable to metal [31]. Unlike zirconia materials, in high-performance polymers separation of the part from the connector does not occur by undergoing plastic deformation.

In a study by Taufall et al. of the fracture resistance of fixed prostheses prepared with PEEK substructure composite resin, it was determined that all the fixed prostheses showed sufficient fracture resistance to expected biting force [32]. In another study, the fracture resistance of PEEK substructure material was found to be suitable for clinical use [33].

Luft et al. reported that implant-supported 3-unit bridge restoration designs using zirconia material with different connector geometries and the cross-section geometry had a significant effect on the mechanical resistance of implant-supported prosthetic restorations. The results obtained were round shape (1065 N) > ellipsoid shape (1010 N) > square shape (870 N) [26]. Previous studies have reported that variables such as the height of the base and the height of the triangle designed for the connector area affect the fracture resistance of the material [21, 23]. Although a square connector has a wider base, it shows lower fracture resistance, as seen in the current study findings.

There were some limitations to this study. The thermomechanical ageing process that was applied (1.2 Hz, 1.2 × 10^6^ cycles) represents a 6-month period. Longer-term evaluation of the fracture resistance of restoration materials could provide more real results that would be obtained in clinical use. However, it is difficult to obtain definitive results as the in vitro conditions cannot fully simulate factors in the oral environent such as saliva, temperature changes due to food and drink, poor oral hygiene, the effects on implants and restorations of bone and gingival changes, and parafunctional forces. When dental tissue and restorative materials are compared, metal antagonists may concentrate the forces applied on surface points and internal zones.

Another limitation of the study could be said to be that comparisons were only made of the fracture resistance of the substructure materials produced with different connector designs without the application of a veneer layer. Although there is a difference in weight between materials with and without veneer porcelain, researchers have demonstrated that it is the material selection, rather than the prosthetic mass, that affects fracture, stress, and strain results [6]. However, the fracture resistance findings could be compared with those of other studies in literature which had used only substructure materials [8, 9, 17, 25]. Carlos et al. stated that layered and monolithic zirconia in the posterior region showed similar fracture resistance [8]. Although Trinia shows superior characteristics than other polymer-based CAD-CAM materials due to high mechanical properties, it is not recommended for monolithic use because of surface properties and aesthetic problems [34]. In-vitro studies allow standardised conditions to be provided in respect of examining the mechanical resistance of materials. Although some studies have examined samples in bar, disc, or cylinder form to determine mechanical resistance, real substructures were produced in the current study to be able to better reflect reality. However, as each material was produced with different milling techniques, it was not possible to provide complete standardisation. In addition, the occlusal geometry of the samples prepared as a substructure material cannot completely replicate the anatomic form of the tooth. Therefore, there is a need for further in vitro and in vivo studies to be conducted supported by various super-structure materials.

## Conclusions

All the 3-unit substructure materials used in this study were seen to be borderline in respect of resisting the forces which can be formed during chewing. The fracture strength of the materials was seen to be influenced by the connector design, with triangular-shaped connectors showing more satisfactory fracture resistance values. The materials with oval-shaped connector design had the weakest fracture resistance in all the groups. Connectors should be designed by evaluatig the embrasure distance and aesthetic criteria. Zirconia showed the highest resistance in all the connector designs, whereas the lowest resistance values were obtained with PEEK material. Brittle fractures were seen in zirconia and the fracture types in the PEEK and FRC groups were ductile in form.

## Data Availability

The datasets used and/or analysed during this study are available from the corresponding author on reasonable request.

## Abbreviations

CAD/CAM: Computer-Aided Design /Computer-Aided Manufacture
Cr-Co: Chrome cobalt
FRC: Fibre-reinforced composite
FDP: Fixed dental prosthesis
N: Newton
PEEK: Poly ether ether ketone
PAEK: Poly aryl ether ketone
SEM: Scanning electron microscope
SPSS: Statistical Package for the Social Sciences
STL: Standard tesselation language
